# Primary syphilis chancre masquerading as a frenulum tear, the essence of histology: a case report

**DOI:** 10.1093/omcr/omac114

**Published:** 2022-10-22

**Authors:** Matthew C Y Tan, Norman Walford, Kok Kuan Tan

**Affiliations:** Saw Swee Hock School of Public Health, National University of Singapore, Singapore; Innoquest Diagnostic Laboratories, Farrer Park Hospital, Singapore; Dr Tan Medical Center, Singapore

## Abstract

Syphilitic chancres are pathognomonic of primary syphilis and can have many atypical presentations. Early detection of such lesions can prevent complications by linking patients to care early. We report a case of a 45-year-old Chinese men who has sex with men (MSM) presenting with a wound on his penis after masturbation. Initial impressions of a frenulum tear secondary to overzealous masturbation led to a circumcision and frenulectomy. Routine histology done provided an unexpected finding of plasma cell infiltrates suggestive of Syphilis. Serology was done to confirm the diagnosis and patient was treated with oral doxycycline for 2 weeks. This case aims to highlight the importance of routine histology during circumcisions especially because lesions such as syphilitic chancres are not always typical in presentation.

## INTRODUCTION

Syphilis is an infection caused by the *Treponema pallidum* spirochete and has a variety of clinical signs and symptoms. A syphilitic infection is classically thought to evolve through 4 basic stages—Primary, Secondary, Latent and Tertiary with additional qualifiers of Early and Late depending on the duration of the infection [1]. While this system provides a framework for understanding the natural history of a syphilitic infection, the key challenge in diagnosing based on such a system is that clinical signs can be subtle and are often mistaken as other less sinister ailments. The traditional teaching of a painless ulcer with a clean base for example tends to be an exception than the rule with many primary syphilitic lesions having many different and atypical presentations [2]. This is the main reason that it is known as the ‘Great Mimicker’ and has gone undetected despite advances in screening methods [3]. Furthermore, because many of the signs in a Primary and Secondary Syphilis resolve spontaneously, patients seldom attend follow-up clinic sessions [4]. Underpinning the importance of early detection of Syphilis is that left untreated, Syphilis progresses into 3 major life-threatening syndromes—Late Neurosyphilis. Cardiovascular Syphilis and Late Benign gummas [5]. Certain populations such as the MSM are known to have a higher prevalence of Syphilis compared with the heterosexual population [6].

Circumcision is the process of partially or completely removing the foreskin and is one of the most common surgical procedures for men [7]. Indications for circumcision include religious/cultural purposes, phimosis, balanoposthitis and risk reduction of Human Immunodeficiency Virus (HIV) transmission [8]. There is much debate about the utility of routine histology of the excised prepuce in circumcisions for medical indications and the extent to which such an investigation changes the overall management.

We report the clinical findings of a patient presenting with wound at the frenulum of the penis noticed immediately after masturbation, which was more in keeping with a traumatic wound, based on the patients’ history, that was eventually confirmed to be a Primary Syphilis infection.

## CASE PRESENTATION

A 45-year-old Chinese man presented to the clinic with a wound at the ventral surface of the penis. Patient reported that he noticed the wound after he masturbated a few days ago. He reported that he felt a sudden pain as he was masturbating and noticed a wound around the frenulum. Patient also complained of pain around the wound especially during erections.

Patient has an allergy to Penicillin but no previous medical history or family history of autoimmune diseases. No history of tobacco smoking or alcohol use. Patient is a Men who has Sex with Men (MSM) and engages in predominantly penetrative anal sex. Patient reports that sexual intercourse is usually protected and has 3–5 sexual partners that year. Partners are usually casual, and patient does not know the HIV or Syphilis status of any of his partners. His last Syphilis and HIV test was done 6 months ago and was non-Reactive. Last sexual intercourse was 3 months ago.

Physical examination found a partial thickness tear at the ventral aspect of the penis, midline and extending 5 mm along the longitudinal aspect of the frenulum. Wound was shallow with regular borders. Base of the lesion was yellow with minimal discharge and no surrounding erythema. No inguinal lymphadenopathy and no other lesions were seen in the groin.

**Figure 1:**
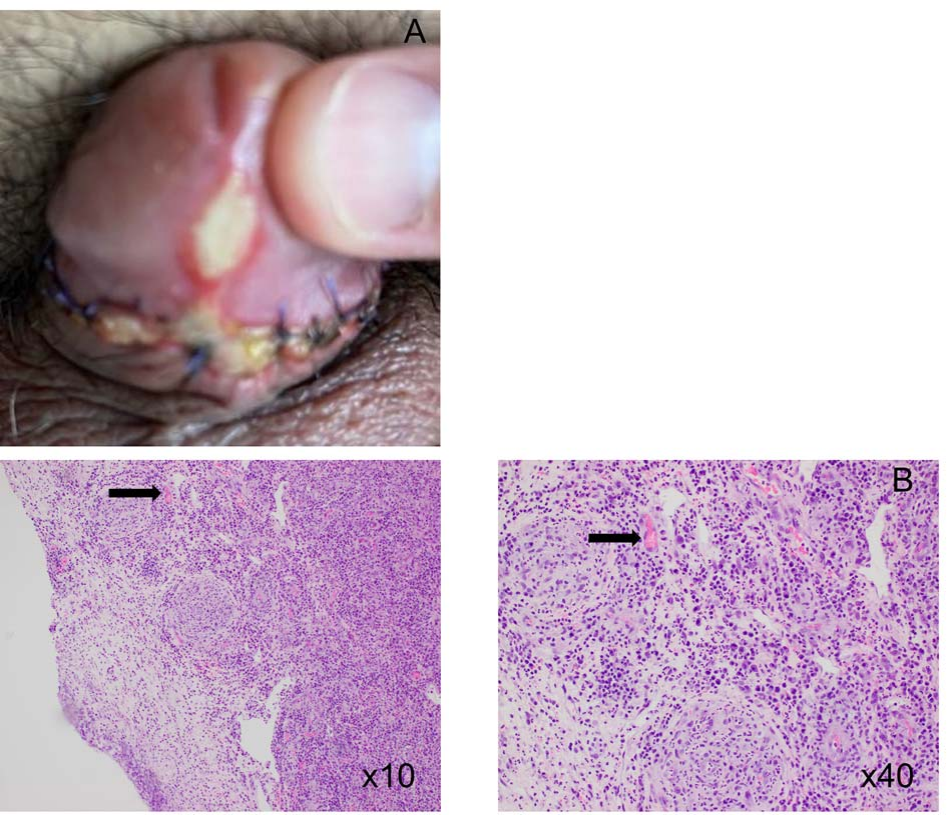
(A) Initial presentation of ulcer; (B) histology of resected foreskin demonstrating presence of plasma cells.

Initial impression was that of a frenulum tear secondary to overzealous masturbation. Patient was offered a frenulectomy to remove the remaining frenulum. Patient opted for a circumcision at the same time as he already had an elective circumcision planned for a few months’ time.

Circumcision was done on the same day of the visit under local anaesthesia. A dorsal slit method was used to remove the foreskin; the remnant frenulum tissue was removed in the same incision. The circumferential wound was closed with 3–0 Vicryl, while the wound at the frenulum was left to heal by secondary intention. Excised tissue including the foreskin and the remnant tissue surrounding the frenulum was sent for histology.

Histology found that the tissue from the remnant tissue of the frenulum to be extensively ulcerated. The ulcer contained a mixture of acute and chronic inflammatory cells with polymorphs towards the surface and a dense underlying mononuclear inflammatory infiltrate including a large number of plasma cells strongly suggestive of a Syphilitic infection.

Syphilis tests were done according to current laboratory protocols. Syphilis serology yielded a Positive Treponemal Test (Syphilis Treponemal Antibody) and Positive for both Non-Treponemal Tests—Rapid Plasmin Reagin (RPR) Titre 1:2 and *T. Pallidum* Particle Agglutination (TPPA).

Given the previous negative Syphilis blood test 6 months ago, a new positive Treponemal and Non-Treponemal blood test result and a wound with plasma cell infiltrates seen on histology, the diagnosis of primary syphilis was made.

Patient was commenced on 2 weeks of doxycycline due to his allergy to penicillin. The patient’s wound healing was uneventful with no secondary infections and completely healed at the end of 2 weeks. A repeat blood test was done 1 month after which demonstrated a decrease in the titre levels of the RPR tests. Follow-up bloods after 6 months demonstrated a Non-Reactive RPR with the Treponemal Antibody and TPPA remaining reactive. The diagnosis was that of Primary Syphilis status post-completed treatment with serological scarring.

## DISCUSSION

We present a case of a man who presented with a wound along the frenulum that occurred after masturbation. Originally thought to be a mechanical frenulum tear from overzealous masturbation, patient elected for a circumcision with a frenulectomy. Routine histology revealed an unexpected finding of plasma cell infiltrates. The patient’s sexual orientation of an MSM, placed him at a higher risk of contracting Syphilis, prompting further testing in the form of Syphilis serology that eventually led to the conclusion that the initial frenulum tear was in fact a primary syphilitic chancre. We hope that this case would highlight the importance of routine histology in circumcision in guiding the diagnostic process.

Currently, there is an ongoing debate on the utility of routine histology for circumcision. Previous studies have compared using clinical signs or histology to diagnose dermatological lesions on the foreskin in patients undergoing circumcision but have yet to come to a consensus on which modality is superior [9, 10].

Most recently, Kerr *et al.* [11] report that clinical diagnosis of lesions in the prepuce correspond to histological diagnosis in up to 80% of cases. Coupled with the lack of evidence associating dermatological lesions of the foreskin with the development of Squamous Cell Carcinoma, these authors are of the opinion that routine histology testing is not required and that clinicians can reliably achieve a diagnosis solely with a physical examination of the area. Additional findings in the study also suggest significant costs saving with the same patient outcome when foregoing a histological investigation.

In a response to these claims, Bunker *et al.* raises an important perspective that a diagnosis need not purely be a clinical or histological diagnosis but a combination of the two. The authors highlighted important shortcomings of the initial study by Kerr *et al.* such as the lack of a uniform control group when making the comparisons between the accuracy of a clinical and histological diagnosis [12]. Indeed, it is further reported by Bunker *et al.* that their centre’s experience up to 45% of the diagnosis of Penile intraepithelial neoplasia was achieved only with a histology with only 8% of the clinicians being confident of the diagnosis without histological confirmation.

From the standpoint of using histology as part of the diagnostic work up instead of solely relying on clinical examination, we agree with that routine histology should be done for circumcision. Syphilis is a prime example of the need for clinical assessment and laboratory investigations to complement each other to provide an accurate diagnosis.

Syphilis is a disease caused by the *T. Pallidum* bacteria and is known as the Great Mimicker as early symptoms can be vague and misdiagnosis based on clinical signs is very common. Primary syphilis is the earliest phase of the infection and is defined by the presence of a painless chancre which usually resolves spontaneously. The situation is similar in secondary Syphilis where a prominent but painless rash erupts and disappears without any intervention. The transient and seemingly indolent nature of the physical manifestations of the initial stages of a Syphilis infection mean that the diagnosis cannot be achieved solely on a physical examination but also requires laboratory investigations. Wound swabs for general bacterial cultures are unable to detect syphilis because the treponemal bacterium does not grow well in regular culture mediums. Although is possible to detect the presence of *T. Pallium* in wounds with dark field microscopy, direct fluorescent antibody testing or polymerase chain reaction, these investigations tend to be labour intensive and not commonly done [13]. Histology of tissue can identify plasma cell infiltrates which is a result of the inflammatory response elicited by the infection and is common in a Primary Syphilitic infection [14].

In the current case, the lesion that the patient presented with was painful and its appearance coincided with a traumatic event (masturbation). The differential diagnoses considered when approaching this case included, trauma, herpes and Syphilis. Although Syphilis was one of the differentials in this case, the lesion reportedly manifested shortly after a traumatic incident of overzealous masturbation and the presence of pain (Syphilitic chancres classically described as being painless) directed us towards considering the wound more traumatic in nature. While there is evidence to suggest that certain at-risk populations such as the MSM group have a higher prevalence of Syphilis, this patient’s sexual orientation was of secondary consideration, and we would like to highlight the process of achieving the diagnosis which was made possible because of routine histology [8]. Without the additional information about the presence of plasma cell infiltrates in the excised tissue provided by routine histology, the diagnosis of Syphilis would have otherwise gone untreated. The early diagnosis and prompt treatment of primary Syphilis in this patient allowed for an early intervention in reducing his risk of developing a more severe form of the disease. This would not have been possible without a combined diagnostic approach of using both clinical examination and laboratory investigation.

Presentations of Syphilis are seldom straightforward; however, the consequences of a missed diagnosis and delay in treatment can allow the disease to progress to Tertiary Syphilis which can be life threatening. We hope that in reviewing this case, we can call to mind the fact that Syphilis chancres are never typical in presentation and the importance of routinely incorporating investigations such as histology in circumcisions to complement clinical assessment.

## AUTHORS’ CONTRIBUTION

All authors contributed equally to the writing and preparation of the manuscript.

## References

[1] O'Byrne P , MacPherson P. Syphilis. Clinical updates. BMJ 2019;365:390–395. 10.1136/bmj.l4159. PMC6598465 31253629

[2] Forrestel AK , Kovarik CL, Katz KA. Sexually acquired syphilis. JAAD 2020;82:1–14. 10.1016/j.jaad.2019.02.073. 30986477

[3] Newman L , Rowley J, Vander Hoorn S, Wijesooriya NS, Unemo M, Low N et al. Global estimates of the prevalence and incidence of four curable sexually transmitted infections in 2012 based on systematic review and global reporting. PLoS One 2015;10:e0143304. 10.1371/journal.pone.0143304. 26646541 PMC4672879

[4] Chen XS . Challenges in responses to syphilis epidemic. Lancet Infect Dis 2017;17:793–4. 10.1016/S1473-3099(17)30327-4. 28701274

[5] LaFond RE , Lukehart SA. Biological Basis for Syphilis. Clin Microbiol Rev 2006;19:29–49. 10.1128/CMR.19.1.29-49.2006. 16418521 PMC1360276

[6] Ong JJ , Fu H, Smith MK, Tucker JD. Expanding syphilis testing: a scoping review of syphilis testing interventions among key populations. Expert Rev Anti-Infect Ther 2018;16:423–32. 10.1080/14787210.2018.1463846. 29633888 PMC6046060

[7] Pintye J , Baeten JM. Benefits of male circumcision for MSM: evidence for action. Lancet Glob Health 2019;7:e388–9. 10.1016/S2214-109X(19)30038-5. 30879496

[8] Yuan T , Fitzpatrick T, Ko NY, Cai Y, Chen Y, Zhao J et al. Circumcision to prevent HIV and other sexually transmitted infections in men who have sex with men: a systematic review and meta-analysis of global data. Lancet Glob Health 2019;7:e436–47. 10.1016/S2214-109X(18)30567-9. 30879508 PMC7779827

[9] Pearce I , Payne SR. Do men having routine circumcision need histological confirmation of the cause of their phimosis or postoperative follow-up? Ann R Coll Surg 2002;84:325–7. 10.1308/003588402760452439. PMC2504154 12398124

[10] McSorley A , Nigam AK. Is routine histology necessary in circumcision? Br J Med Surg Urol 2011;4:148–51. 10.1016/j.bjmsu.2010.10.001.

[11] Kerr L , Hendry J, Crookshanks A, Taylor J. Does routine histology alter management post circumcision? J Clin Urol 2020;13:279–82. 10.1177/2051415819895399.

[12] Bunker C , Kravvas G, Watchorn R, Spencer A, Ong E, Haider A et al. Reply to: ‘Does routine histology alter management post-circumcision?’. J Clin Urol 2022;15:19–22. 10.1177/2051415820982755.

[13] Park IU , Fakile YF, Chow JM, Gustafson KJ, Jost H, Schapiro JM et al. Performance of treponemal tests for the diagnosis of syphilis. Clin Infect Dis 2019;68:913–8. 10.1093/cid/ciy558. 29986091 PMC6326891

[14] Carlson JA , Dabiri G, Cribier B, Sell S. The immunopathobiology of syphilis: the manifestations and course of syphilis are determined by the level of delayed-type hypersensitivity. Am J Dermatopathol 2011;33:433–60. 10.1097/DAD.0b013e3181e8b587. 21694502 PMC3690623

